# 

**Published:** 1913-10-11

**Authors:** 


### CONTENTS.

| | PAGE |
|---|---|
| **Mental Hospitals: Past and Present** ... ... | 25 |
| **The Sterilsation of the Unfit** ... ... ... | 26 |
| **Hospital and Institutional News,** including— ... Absconding Phthisical Patients—Metropolitan Asylums Board and Sanatoria—Dr. Robert Jones on Failures—The Passing of the General Hospital—Cheap Thermometers: A Warning to Institutional Managers—Spirited Protest by a Medical Superintendent | 27 |
| **Four Practical Aspects of the Mental Deficiency Act, 1913** ... ... ... ... By Sir BRYAN DONKIN, M.D., F.R.C.P. | 33 |
| **The Mental Deficiency Act, 1913** ... ... ... The General Scope of its Provisions. | 35 |
| **The Editor's Letter-Box** ... ... ... ... The Lord Mayor and Hospital Saturday | 36 |
| **The Feeble-Minded as Producers** ... ... ... By Miss DENDY, Sec. Society for Care of Feeble-Minded. | 37 |
| **The Work of the Darenth Industrial Colony, Dartford** ... ... ... ... ... ... | 39 |
| **Registered Hospitals and Asylums** ... ... By Dr. GILMOUR, St. Luke's Hospital. | 42 |
| **Mental Research and Government Grants** ... By JOSEPH SHAW BOLTON, D.Sc., M.D., F.R.C.P. | 43 |
| **Statistical Tables of Recoveries** ... ... ... The Value of Recovery Rates. | 46 |
| **Cooking and Catering in Mental Hospitals** ... By J. FRANCIS DIXON, M.D. | 47 |
| **Trade Unionism in Mental Hospitals** ... ... | 49 |
| **The Last Days for Entering Insurance on Special Terms** ... ... ... ... ... | 51 |
| **The Gas Exhibition** ... ... ... ... ... | 52 |
| **The Threatened Strike of Asylum Nurses** ... | 54 |
| **The Institutional Worker** ... ... (Suppl.) | 1 |
| **Our Bureau of Information** ... ... ,, | 2 |
| **Books Received** ... ... ... ... ,, | 3 |
| **Institutional Needs** ... ... ... ,, | 3 |
| **Buildings Contemplated and in Progress** ,, | 3 |

